# 3D collagen migration patterns reveal a SMAD3-dependent and TGF-β1-independent mechanism of recruitment for tumour-associated fibroblasts in lung adenocarcinoma

**DOI:** 10.1038/s41416-022-02093-x

**Published:** 2022-12-26

**Authors:** Yago Juste-Lanas, Natalia Díaz-Valdivia, Alejandro Llorente, Rafael Ikemori, Alejandro Bernardo, Marselina Arshakyan, Carlos Borau, Josep Ramírez, José Carlos Ruffinelli, Ernest Nadal, Noemí Reguart, José M. García-Aznar, Jordi Alcaraz

**Affiliations:** 1 Department of Mechanical Engineering, Aragón Institute of Engineering Research (I3A), University of Zaragoza, 50018 Zaragoza, Spain; 2 Department of Biochemistry and Molecular and Cellular Biology, University of Zaragoza, 50009 Zaragoza, Spain; 3 Unit of Biophysics and Bioengineering, Department of Biomedicine, School of Medicine and Health Sciences, Universitat de Barcelona, 08036 Barcelona, Spain; 4 Institute for Bioengineering of Catalonia (IBEC), The Barcelona Institute for Science and Technology (BIST), 08028 Barcelona, Spain; 5 Pathology Service, Hospital Clínic de Barcelona, 08036 Barcelona, Spain; 6 Thoracic Oncology Unit, Hospital Clinic Barcelona, 08036 Barcelona, Spain; 7 Department of Medical Oncology, Catalan Institute of Oncology, L’Hospitalet de Llobregat (Barcelona), 08908 Barcelona, Spain; 8 Preclinical and Experimental Research in Thoracic Tumors (PrETT) group, Oncobell Program, Bellvitge Biomedical Research Institute (IDIBELL), L’Hospitalet de Llobregat (Barcelona), 08908 Barcelona, Spain; 9 Institut d’Investigacions Biomèdiques August Pi i Sunyer (IDIBAPS), Barcelona, 08036 Spain

**Keywords:** Non-small-cell lung cancer, Mesenchymal migration, Non-small-cell lung cancer, Cancer microenvironment

## Abstract

**Background:**

The TGF-β1 transcription factor SMAD3 is epigenetically repressed in tumour-associated fibroblasts (TAFs) from lung squamous cell carcinoma (SCC) but not adenocarcinoma (ADC) patients, which elicits a compensatory increase in SMAD2 that renders SCC-TAFs less fibrotic. Here we examined the effects of altered SMAD2/3 in fibroblast migration and its impact on the desmoplastic stroma formation in lung cancer.

**Methods:**

We used a microfluidic device to examine descriptors of early protrusions and subsequent migration in 3D collagen gels upon knocking down SMAD2 or SMAD3 by shRNA in control fibroblasts and TAFs.

**Results:**

High SMAD3 conditions as in shSMAD2 fibroblasts and ADC-TAFs exhibited a migratory advantage in terms of protrusions (fewer and longer) and migration (faster and more directional) selectively without TGF-β1 along with Erk1/2 hyperactivation. This enhanced migration was abrogated by TGF-β1 as well as low glucose medium and the MEK inhibitor Trametinib. In contrast, high SMAD2 fibroblasts were poorly responsive to TGF-β1, high glucose and Trametinib, exhibiting impaired migration in all conditions.

**Conclusions:**

The basal migration advantage of high SMAD3 fibroblasts provides a straightforward mechanism underlying the larger accumulation of TAFs previously reported in ADC compared to SCC. Moreover, our results encourage using MEK inhibitors in ADC-TAFs but not SCC-TAFs.

## Introduction

Lung cancer is the leading cause of cancer mortality worldwide, with a 5-year survival rate of ~19% [1]. Histologically, non-small cell lung cancer (NSCLC) is diagnosed in ~85% of lung cancer patients, and is classified into adenocarcinoma (ADC; ~50%), squamous cell carcinoma (SCC; ~40%) and other less frequent subtypes [2]. SCC tumours are strongly associated with smoking and are commonly located in proximal airways, whereas ADC typically arise in distal pulmonary sites [2]. Although both ADC and SCC are epithelial in origin, it is increasingly recognised that the desmoplastic/fibrotic stroma rich in tumour-associated fibroblasts (TAFs, also referred to as cancer-associated fibroblasts or CAFs), play a key role in tumour progression and therapy resistance [3, 4]. Accordingly, there is growing interest in understanding the aberrant behaviour of fibroblasts in solid tumours [5].

Most lung TAFs exhibit an activated/myofibroblast-like phenotype [6, 7], which is roughly characterised by the intracellular expression of α-smooth muscle actin (α-SMA) and the abundant extracellular deposition of fibrillar collagens [3]. Transforming growth factor-β1 (TGF-β1) is the most potent fibroblast activator known to date and is frequently upregulated in NSCLC. Moreover, both TGF-β1 and TAF activation markers are associated with poor prognosis in NSCLC [7, 8]. Intriguingly, we recently reported that TAF activation and associated fibrosis is higher in ADC compared to SCC, owing to the larger epigenetic repression of the important pro-fibrotic transcription factor SMAD3 of the canonical TGF-β pathway selectively in SCC-TAFs, caused by their increased exposure to cigarette smoke particles. We also showed that the epigenetic repression of SMAD3 elicited a compensatory increase in the expression and activity of its closely related homologue SMAD2 in SCC-TAFs, which is weakly associated with fibrosis [9]. Consistently, ADC-TAFs but not SCC-TAFs exhibited a positive response to the antifibrotic drug nintedanib in culture [10], thereby mimicking the therapeutic benefits reported by nintedanib in ADC but not in SCC patients in the LUME-1 clinical trial [11]. Likewise, both the lower expression of fibrosis markers and the poor nintedanib response of SCC-TAFs could be reproduced in normal fibroblasts upon knocking down SMAD3 with shRNA, whereas knocking down SMAD2 had opposite effects, supporting that shSMAD2 and shSMAD3 fibroblasts exhibit ADC-like and SCC-like phenotypes, respectively [9, 12].

SMADs 2 and 3 (referred to as SMAD2/3 thereafter) are direct mediators of canonical TGF-β1 signalling and exhibit some overlapping functions; however, they also regulate distinct processes, as reported in knock-out mice [13]. In fibroblasts, SMAD2/3 not only differentially regulate fibrosis and response to antifibrotic drugs [9, 14] but they may also control cell migration, which may be relevant for the formation of the fibrotic tumour microenvironment (TME). However, our knowledge of how SMAD2/3 control cell migration is very limited [15], and their impact on the formation of the desmoplastic TME in lung cancer is unknown. Moreover, the few available fibroblast-specific analyses of migration regulation by SMAD2/3 were performed in two dimension (2D) or transwells [15, 16], whereas fibroblast migration in vivo occurs within a three-dimensional (3D) microenvironment rich in type I collagen [17]. To address this gap of knowledge, we knocked down either SMAD2 or SMAD3 in primary human pulmonary fibroblasts as surrogate models of ADC-TAFs or SCC-TAFs, respectively. Fibroblasts were cultured in dense 3D collagen gels in the absence or presence of TGF-β1 to mimic the progression of the desmoplastic tumour stroma [18], and the formation of pro-migratory protrusions and subsequent migration was analysed. For this purpose, 3D cultures were prepared within a microfluidic device to assess a panel of biophysical descriptors of protrusions and migration by multidimensional microscopy, and key findings were validated with ADC-TAFs.

## Methods

### Patient-derived tissue samples and pulmonary fibroblasts

Primary fibroblasts were previously obtained from a cohort of 20 NSCLC surgical patients [6]. Fibroblasts were derived from either tumour or patient-matched uninvolved pulmonary tissue (referred to as control fibroblasts thereafter) using protocols approved by the Ethics Committees of the Hospital Clinic de Barcelona and the Universitat de Barcelona. Selected patients were male, chemo-naïve, Caucasian, >55 years old and current/former smokers. Tumour tissue samples for histological analysis were obtained from the Hospital de Bellvitge (10 ADC, 9 SCC) with the approval of the Ethics Committee. The study was performed in accordance with the Declaration of Helsinki and written informed consent was obtained from all patients. Further clinical characteristics are shown in Supplementary Table 1.

### Histologic analysis

Tumour samples were processed as described [9], counterstained with haematoxylin and stained for either cleaved microtubule-associated protein 1 light chain 3 (LC3A) (#Ap1805a, Abgent), which is largely negative in fibroblasts [19], eosin or α-SMA as reported [6]. Fibroblast nuclear density was assessed by image analysis of haematoxylin staining using the QuPath software [20] under the guidance of our pathologist (JR). Further details are provided in Supplementary Materials.

### 2D cell culture and fibroblast immortalisation

Control fibroblasts and ADC-TAFs from randomly selected patients (#5, #13, #37) were immortalised with hTERT as reported [9]. Unless otherwise indicated, all fibroblast experiments were performed in culture medium containing serum-free high-glucose (4.5 g/l) DMEM supplemented with 1% insulin–transferrin–selenium (ITS) and antibiotics as described [10]. In some experiments, fibroblasts were stimulated with 2.5 ng/ml recombinant human TGF-β1 (Miltenyi Biotec) at different time points as indicated, which is similar to the average TGF-β1 concentration reported in the bronchoalveolar lavage fluid of lung cancer patients [21].

### SMAD2 and SMAD3 knock down with shRNA and siRNA

SMAD2 or SMAD3 were stably knocked down in immortalised primary control fibroblasts and ADC-TAFs with lentiviral vectors derived from Sigma MISSION collection as reported [9]. A nonmammalian targeting shRNA vector was used as control (shControl). Alternatively, SMAD3 was transiently knocked down by siRNA as described [9]. Further details are provided in Supplementary Materials.

### qRT-PCR

RNA extraction and reverse transcription were conducted as reported [10, 22]. *SMAD2*/*3* and *MMP1* mRNA levels were assessed using specific primers, with *ACTB* or *POLR2A* as endogenous controls, respectively. Further details are provided in Supplementary Materials.

### Western blot (WB) analysis

WB analysis of SMAD2/3 and Erk1/2 was conducted as described [6, 9], using primary antibodies against total SMAD2/3 (#3102, Cell Signaling), pSMAD2 (#3104, Cell Signaling), pSMAD3 (#07-1389, Merck Millipore), Erk1/2, pErk1/2 (#9102 and #9101; Cell Signaling Technology), β-actin (#A1978, Sigma-Aldrich) and α-tubulin (#2144; Cell Signaling Technology). The latter two were used as loading controls. Additional details are provided in Supplementary Materials.

### Fabrication of the microfluidic device

Microfluidic devices were fabricated as described [23], using photomasks as previously reported [24, 25]. In brief, masks were used to fabricate positive 300 µm high SU8 masters (Stanford University). Polydimethylsiloxane (PDMS) (Sylgard 184, Dow Corning) was mixed at a 10:1 weight ratio of base to curing agent and poured on the SU8 master until the desired thickness (4 mm) was obtained. The PDMS solution was cured in an oven, cut out and removed from the wafer, perforated and autocleaved. PDMS microdevices were plasma-bonded to 35 mm glass-bottom petri dishes (Ibidi) and coated with 1 mg/ml poly-d-lysine (PDL) (Sigma-Aldrich) to enhance surface-collagen gel attachment. The geometry of the microdevice was based on [26] and included a 300 µm high central chamber to allocate the 3D collagen culture and two parallel liquid channels located on each side of the central chamber that were in direct contact with the gel for hydration and transport of nutrients and other factors [25]. Further details are provided in Supplementary Materials.

### 3D collagen cell culture within the microfluidic device

Collagen hydrogels were prepared as reported [23, 25]. Briefly, type I collagen solution (BD Bioscience) was mixed with DPBS (Thermo Fisher Sci.) and neutralised with NaOH (Sigma-Aldrich) to pH 7.4. For 3D culture experiments, cells suspended in culture medium were mixed with the collagen solution to a final concentration of 4 mg/ml and cell dilution of 2 × 10^5^ cells/ml, which enables local matrix remodelling but not global gel contraction [17]. The collagen and cells solution were loaded into the central chamber of the microfluidic device using the auxiliary inlet channels and attached to it through surface tension. The device containing the collagen and cells solution was placed into an incubator to allow collagen polymerisation for 30 min. Next, the 3D culture was hydrated with culture medium and kept in the incubator before experiments.

### Analysis of the protrusions of single fibroblasts in 3D collagen cultures

Protrusion analysis was performed by adapting our previous protocol [27, 28]. Fibroblasts were kept with culture medium with or without 2.5 ng/ml TGF-β1 for 72 h in 2D culture, trypsinised and used to prepare 3D collagen cultures within the microfluidic device. Protrusions were imaged after collagen polymerisation and up to 4 h based on previous observations [17] as reported [27, 28], using a phase contrast Nikon Eclipse Ti-E inverted microscope (Nikon, Japan) provided with an incubator. Imaging was conducted at least 100 µm away from the glass and PDMS surface to avoid potential edge effects [29]. The whole gel thickness (300 μm) was imaged at 5 μm intervals (*Z* axis) every 5 min at ×200 magnification with a ×20 objective (CFI Plan Fluor ELWD ADM, NA 0.45; Nikon), eliciting 49 time points and 2940 images/fibroblast. Best *Z* plane was chosen for each image, and both the cell body and the protrusions of randomly selected fibroblasts were manually outlined [27, 28]. The aspect ratio was computed as major axis/minor axis as reported [30] (Supplementary Fig. 1). A panel of descriptors was analysed for each “mother” protrusion that stemmed from the cell body (referred to as protrusions thereafter) [27, 31], including length, number, and protrusion and retraction growth rates, using in-house MATLAB (MathWorks) algorithms. For each fibroblast, the absolute values for the protrusion growth and retraction rates were averaged to indicate protrusion evolution rate. A total of 4 fibroblasts/condition were examined from 3 independent experiments, which required analysing >8000 images in total.

### Analysis of the migration of single fibroblasts in 3D collagen cultures

Random fibroblast migration was analysed using our previous protocols [24, 25, 32]. Fibroblasts were kept with culture medium with or without 2.5 ng/ml TGF-β1 for 48 h in 2D culture, trypsinised and used in 3D collagen cultures within the microfluidic device. 3D cultures were kept with or without TGF-β1 for 24 h within the device, and subsequently imaged for 24 h with a phase contrast inverted microscope provided with an incubator. Images were acquired every 20 minutes in a manually selected *Z* plane at ×100 magnification using a ×10 air objective (CFI Plan Fluor DLL, NA 0.30, WD 16.0 mm; Nikon) as described [24, 33]. Imaging was conducted at least 100 µm away from the glass and PDMS surface to avoid potential artifacts. The trajectories of randomly selected fibroblasts were tracked and used to compute a panel of migration descriptors with in-house MATLAB algorithms [24, 34], including average cell speed, net displacement and cell persistence. On average, 35 fibroblasts were analysed for each device, and 190 per condition.

### Boyden Transwell migration assay

Fibroblasts migration was performed using the Transwell Boyden assay as reported [35]. In brief, fibroblasts were maintained for 3 days in serum-free culture medium with or without 2.5 ng/ml TGF-β1 before seeding them on Transwell inserts. Culture medium alone or supplemented with 2.5 ng/ml TGF-β1 was added to the lower Transwell compartment, and cells that migrated into the lower insert membrane side after 16 h were fixed, stained with crystal violet and imaged by phase-contrast microscopy with a ×10 objective. Migration was assessed as percentage of positively stained image area with Image J. In some experiments, fibroblasts were treated with 100 nM Trametinib (Selleckchem).

### Fibroblast number density

Cell number density of TAFs in 2D cultures was assessed as reported [6]. In brief, TAFs were cultured in serum-free medium with or without 2.5 ng/ml TGF-β1 for 5 days and their nuclei were stained with Hoechst 33342 (Molecular Probes) and imaged with a ×10 objective. Number density in the same 3D cultures used for 3D migration analysis were assessed by manually counting cells imaged at the end of the experiment. For each experiment, number density was determined as the average cell density/image.

### Statistical analysis

Two-group comparisons were performed with either two-tailed Student’s *t* test or Mann–Whitney test for non-parametric data (GraphPad Prism v9.0.). Statistical significance was assumed at *p* < 0.05. All experiments were conducted at least in triplicates (≥3 microfluidic devices). All data shown are mean ± s.e.m.

## Results

### The relative differences in SMAD2/3 expression between ADC-TAFs and SCC-TAFs can be mimicked through shRNA in control pulmonary fibroblasts

**Fig. 1:**
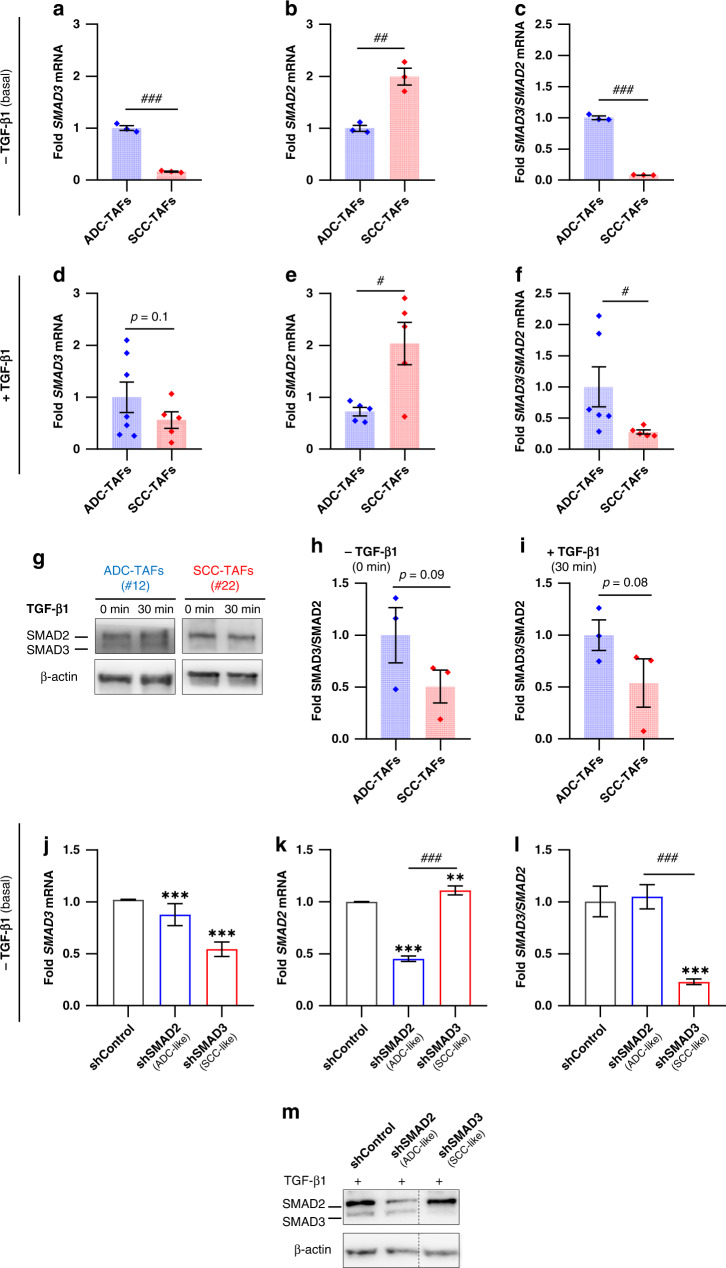
Genetic models to mimic SMAD2/3 alterations in patient-derived ADC-TAFs and SCC-TAFs. **a**–**c** Fold *SMAD3* (**a**) and *SMAD2* (**b**) mRNA and corresponding ratio (**c**) in primary lung TAFs cultured in 2D for 3 days in basal conditions (i.e. without exogenous TGF-β1) (3 ADC, 3 SCC). **d**–**f** Fold *SMAD3* (**d**) and *SMAD2* (**e**) mRNA and corresponding ratio (**f**) in primary lung TAFs cultured in 2D for 3 days in the presence of 2.5 ng/ml TGF-β1 (7 ADC, 5 SCC). **g** Representative Western blot for total SMAD2, SMAD3 and β-actin of ADC-TAFs and SCC-TAFs from randomly selected patients at 0 min or 30 min after stimulation with TGF-β1. **h**, **i** Densitometry analysis of total SMAD3/SMAD2 ratio in TAFs from randomly selected patients (3 ADC, 3 SCC) at 0 min (h) or 30 min (i) after stimulation with TGF-β1. **j**–**l** Fold *SMAD3* (**j**), *SMAD2* (**k**) and *SMAD3*/*SMAD2* mRNA ratio (**l**) of shControl, shSMAD2 and shSMAD3 control fibroblasts from patient #5 cultured in 2D for 3 days in basal conditions. **m** Representative Western blot for total SMAD2, SMAD3 and β-actin of shControl, shSMAD2 and shSMAD3 control fibroblasts (#5) in basal conditions. Error bars represent mean ± s.e.m. Each dot corresponds to a different patient (**a**–**i**). ^#^*p* < 0.05; ^##^*p* < 0.01; ^###^*p* < 0.005 comparing either ADC-TAFs and SCC-TAFs or shSMAD2 and shSMAD3. **p < 0.01; ***p < 0.005 with respect to shControl. Statistical comparisons were done using Student’s *t* test.

To characterise the differences in SMAD2/3 in ADC-TAFs and SCC-TAFs in more detail, we examined SMAD2/3 expression in TAFs from randomly selected patients in the absence or presence of TGF-β1 and without normalising by patient-matched control fibroblasts as in our previous study [9]. In basal conditions (i.e. absence of TGF-β1) *SMAD3* mRNA was significantly higher in ADC-TAFs than SCC-TAFs (Fig. 1a), whereas *SMAD2* mRNA exhibited the opposite trend (Fig. 1b), eliciting a markedly higher *SMAD3*/*SMAD2* mRNA ratio in ADC-TAFs (Fig. 1c). These histotype differences were maintained in response to TGF-β1 (Fig. 1d–f) and are in agreement with the larger epigenetic repression of *SMAD3* in SCC-TAFs [9]. Similar histotype differences in SMAD3/SMAD2 ratio were found at the protein level (Fig. 1g–i and Supplementary Fig. 2), and collectively reveal that ADC-TAFs exhibit high *SMAD3* mRNA and SMAD3/SMAD2 expression ratio, whereas SCC-TAFs exhibit high *SMAD2* mRNA and lower SMAD3/SMAD2 expression ratio. Consistently, we previously showed that the response to TGF-β1 is dominated by the activation through phosphorylation of either SMAD3 in ADC-TAFs or SMAD2 in SCC-TAFs, concomitantly with a higher expression of fibrosis markers in ADC-TAFs compared to SCC-TAFs [9].

To model the SMAD2/3 differences observed in TAFs, we stably knocked down SMAD2 or SMAD3 by shRNA in control fibroblasts derived from uninvolved pulmonary tissue of a randomly selected surgical lung cancer patient (#5). In basal conditions, shSMAD2 fibroblasts exhibited higher *SMAD3* mRNA (Fig. 1j) and protein expression (Fig. 1m) than shSMAD3 fibroblasts as in ADC-TAFs. Conversely, shSMAD3 fibroblasts exhibited the largest *SMAD2* mRNA (Fig. 1k) and protein levels (Fig. 1m) as in SCC-TAFs, which elicited the largest *SMAD3*/*SMAD2* mRNA ratio in shSMAD2 fibroblasts (Fig. 1l). In further agreement with TAFs, the response of shSMAD2 fibroblasts to exogenous TGF-β1 was dominated by the activation through phosphorylation of SMAD3 as in ADC-TAFs, whereas that of shSMAD3 fibroblasts was dominated by the phosphorylation of SMAD2 (Supplementary Fig. 2) as in SCC-TAFs [9]. Accordingly, and in agreement with previous studies [9, 10], shSMAD2 and shSMAD3 fibroblasts were used as ADC-like and SCC-like models henceforth, respectively.

### Analysis of protrusions in 3D collagen gels reveals that high SMAD3 conditions as in ADC-TAFs primes fibroblasts for migration in the absence of exogenous TGF-β1

**Fig. 2:**
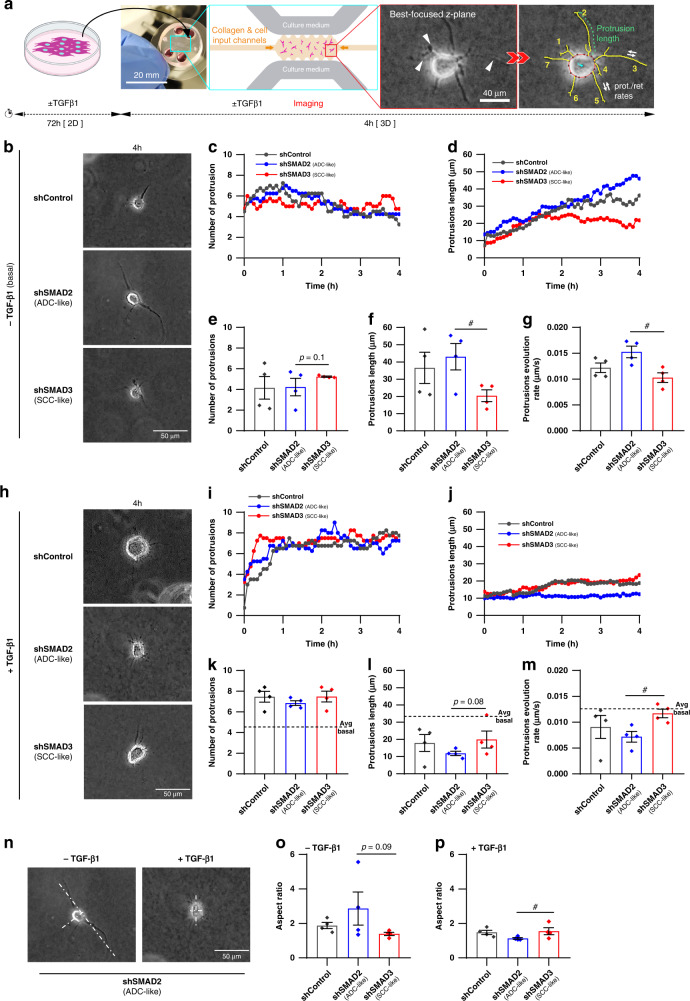
Impact of altered SMAD2/3 in 3D collagen protrusions of lung fibroblasts and ADC-TAFs with or without exogenous TGF-β1. **a** Outline of the microdevice-based analysis of protrusions (0–4 h) of single fibroblasts cultured in dense 3D collagen gels. **b** Representative phase contrast images of single control fibroblasts (#5) for each group (shControl, shSMAD2, shSMAD3) cultured in 3D collagen gels within the microdevice for 4 h in basal conditions (−TGF-β1). Scale bar, 50 μm. Representative images at other time points are shown in Supplementary Fig. 3A, B. **c**, **d** Time-course of the average number of protrusions (**c**) and protrusion length (**d**) of 4 fibroblasts for each group cultured in 3D in basal conditions. **e**–**g** Number of protrusions (**e**), protrusion length (**f**) and evolution rate (**g**) averaged for 4 fibroblasts per group within the last 1 h (3–4 h) of the experimental time-window (**e**, **f**) or the full experiment (0–4 h) (**g**) in basal conditions (shown as bars). Each dot indicates the average of a single fibroblast henceforth. **h** Representative phase contrast images of single control fibroblasts (#5) for each group (shControl, shSMAD2, shSMAD3) cultured in 3D collagen gels within the microdevice for 4 h in the presence of TGF-β1. Scale bar, 50 μm. Representative images at other time points are shown in Supplementary Fig. 3A, B. **i**, **j** Time-course of the average number of protrusions (**i**) and protrusion length (**j**) of 4 fibroblasts for each group cultured in 3D in the presence of TGF-β1. **k**–**m** Number of protrusions (**k**), protrusion length (**l**) and evolution rate (**m**) averaged for 4 fibroblasts per group as in **e**–**g** in the presence of TGF-β1. **n** Representative phase contrast images of the aspect ratio analysis of single shSMAD2 fibroblasts cultured in 3D collagen gels for 4 h without or with TGF-β1. Scale bar, 50 μm. Further details on the assessment of the aspect ratio shown in Supplementary Fig. 1. **o**, **p** Aspect ratio averaged for 4 fibroblasts per group at the end of the experimental time-window (4 h) in basal conditions (**o**) or in the presence of TGF-β1 (**p**). Error bars represent mean ± s.e.m. Each dot corresponds to the average of a different fibroblast (**e**–**g**, **k**–**m**, **o**, **p**) examined within 3 independent microdevices. ^#^*p* < 0.05 comparing shSMAD2 and shSMAD3. Other p-values comparing to shControl. Statistical comparisons were done using Student’s *t* test.

Membrane protrusions are pointed as critical regulators of cell migration in 3D [31, 36]. We used a microdevice-based assay [27] to monitor a panel of protrusion descriptors in single fibroblasts embedded in a dense 3D collagen gel within the first 4 h as outlined in Fig. 2a. Protrusion analysis was limited to 4 fibroblasts per condition owing to the large number of images involved in each fibroblast analysis. All fibroblasts initially exhibited a round morphology with a dendritic network of protusions (Fig. 2b, h and Supplementary Fig. 3A, B). In basal conditions, the average number of protrusions fluctuated around 4–7, with a modest increase within the first 1 h followed by a slow decline in shSMAD2 (ADC-like) and shControl fibroblasts down to 4, whereas they remained stable around 5 in shSMAD3 (SCC-like) fibroblasts (Fig. 2c, e). In contrast, protrusion length increased with time selectively in shSMAD2 fibroblasts up to 50 μm, whereas it reached a plateau in the last 1–2 h that was the lowest in shSMAD3 fibroblasts (Fig. 2d). Accordingly, protrusion number and length were averaged within the last hour (3–4 h) henceforth and were found consistently higher in shSMAD2 compared to shSMAD3 fibroblasts in terms of length (Fig. 2f) and growth/retraction evolution rate (Fig. 2g), with average values of ~40 μm and 0.015 μm/s, respectively. Conversely, protrusion number was lower in shSMAD2 than shSMAD3 fibroblasts with marginal significance (Fig. 2e).

Unlike basal conditions, all TGF-β1-preactivated fibroblasts exhibited a low initial number of protrusions (1-3) that increased within the first 1 h up to 7, although at different rates, in which shSMAD3 fibroblasts were the fastest (Fig. 2i). In contrast, protrusion length increased over time at a much lower rate than in basal conditions, particularly in shSMAD2 fibroblasts, which attained a protrusion length of ~10 μm (Fig. 2j). Globally, TGF-β1 elicited an average ≥50% increase of protrusion number compared to basal conditions (Fig. 2k and Supplementary Fig. 3C), whereas it reduced both protrusion length and evolution rate by >50% in shSMAD2 conditions, and these descriptors remained stable in shSMAD3 fibroblasts (Fig. 2l, m and Supplementary Fig. 3D, E), suggesting that shSMAD3 fibroblasts are poorly responsive to TGF-β1. Since longer and fewer protrusions have been previously associated with enhanced migration [31], these results suggest that shSMAD2 (ADC-like) fibroblasts may be primed for migration selectively in basal conditions. Consistently, the aspect ratio at 4 h, which is indicative of polarisation along a major protrusion (Supplementary Fig. 1) and has been positively associated with migration [36], was the highest in shSMAD2 fibroblasts in basal conditions (Fig. 2n, o), whereas it was the lowest upon TGF-β1 preactivation (Fig. 2p).

### Analysis of migration in 3D collagen gels confirms that high SMAD3 as in ADC-TAFs enhances migration selectively in basal conditions

**Fig. 3:**
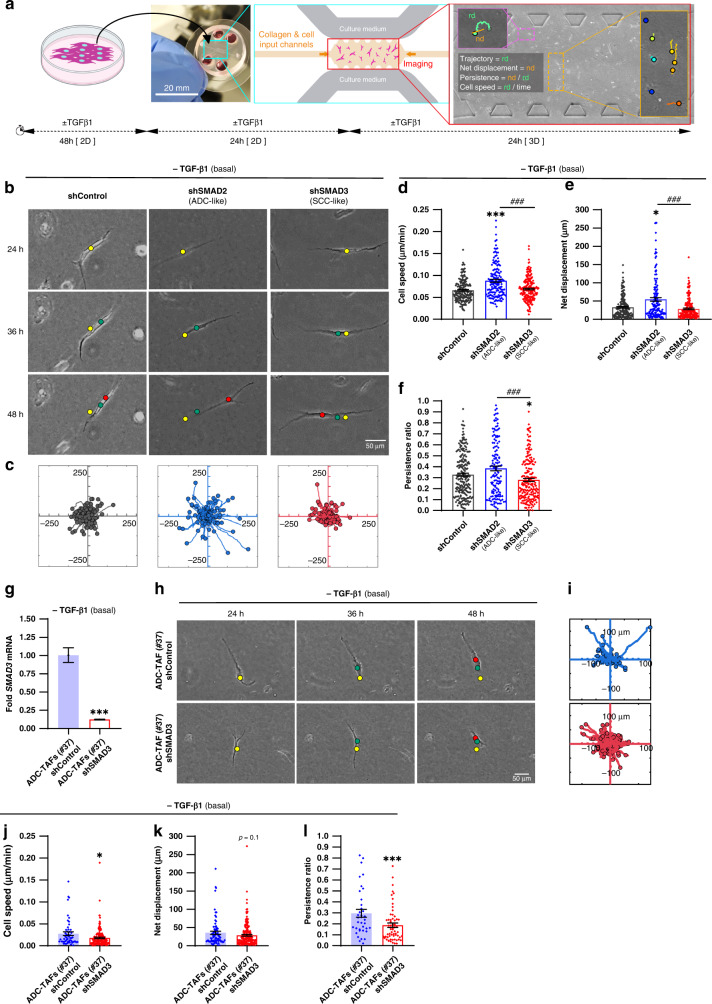
Impact of altered SMAD2/3 in 3D collagen migration in lung fibroblasts and ADC-TAFs in basal conditions (no exogenous TGF-β1). **a** Outline of the microdevice-based analysis of migration (24–48 h) of single fibroblasts cultured in dense 3D collagen gels. **b** Representative phase contrast images of single control fibroblasts (#5) for each group (shControl, shSMAD2, shSMAD3) cultured in 3D collagen gels within the microdevice in basal conditions during the experimental time-window (24–48 h). Yellow, green and red dots indicate the position of the fibroblast centre at 24, 36 and 48 h, respectively. Scale bar, 50 μm. **c** Trajectory maps corresponding to the tracking of the centre of fibroblasts from each group in basal conditions throughout the experimental time-window. The starting of each fibroblast was shifted to the origin of coordinates for clarity here and thereafter. **d**–**f** Average cell speed (**d**), net displacement (**e**) and persistence ratio (**f**) in basal conditions of single control fibroblasts (#5) for each group (shControl, shSMAD2, shSMAD3) gathered from 3 independent microdevices per condition (shown as bars). Each dot indicates the average of a single fibroblast henceforth. Note that persistence values range between 0 and 1, where 1 indicates migration without changing direction [30]. **g** Fold *SMAD3* mRNA of shControl and shSMAD3 ADC-TAFs (#37) cultured as in Fig. 1j. **h** Representative phase contrast images of single shControl or shSMAD3 ADC-TAFs (#37) cultured as in **b**. Yellow, green and red dots indicate the position of the fibroblast centre at 24, 36 and 48 h, respectively. Scale bar, 50 μm. **i** Trajectory maps corresponding to the tracking of the centre of ADC-TAFs from each group in basal conditions throughout the experimental time-window as in **c**. **j**–**l** Average cell speed (**j**), net displacement (**k**) and persistence ratio (**l**) of single ADC-TAFs (#37) for each group (shown as bars) cultured as in **b**. Error bars represent mean ± s.e.m. ^###^*p* < 0.005 comparing shSMAD2 and shSMAD3. *p < 0.05; ***p < 0.005 with respect to shControl or comparing shControl and shSMAD3 ADC-TAFs. Statistical comparisons were done using Student’s *t* test or Mann–Whitney (**d**–**f**). Mean values correspond to three independent microdevices.

Next, we adapted the microfluidic device assay to analyse single fibroblast migration within 24–48 h in 3D culture as outlined in Fig. 3a. All fibroblasts exhibited the archetypical elongated morphology found in histologic sections [6, 17] (Fig. 3b). In agreement with protrusion analysis, shSMAD2 (ADC-like) fibroblasts exhibited significantly enhanced migration in basal conditions, as illustrated by their larger trajectories (Fig. 3c) and significantly higher values of all migration descriptors compared to shSMAD3 and shControl fibroblasts (Fig. 3d–f), with an average speed of ~0.10 μm/min (Fig. 3d), total net displacement of ~50 μm (Fig. 3e) and directional persistence of ~0.4 (Fig. 3f) that were ~30% (speed), ~90% (displacement) and ~40% (persistence) larger than the corresponding values of shSMAD3 fibroblasts. To validate our observations, we analysed ADC-TAFs from patient #37 upon knocking down SMAD3 with shRNA (Fig. 3g) in the absence of exogenous TGF-β1. Although migration descriptors in ADC-TAFs (#37) (Fig. 3h–l) were lower than those found in control fibroblasts (Fig. 3d–f), shControl ADC-TAFs exhibited in average a larger effective speed (Fig. 3j), net displacement (Fig. 3k) and persistence (Fig. 3l) than shSMAD3 ADC-TAFs (#37).

**Fig. 4:**
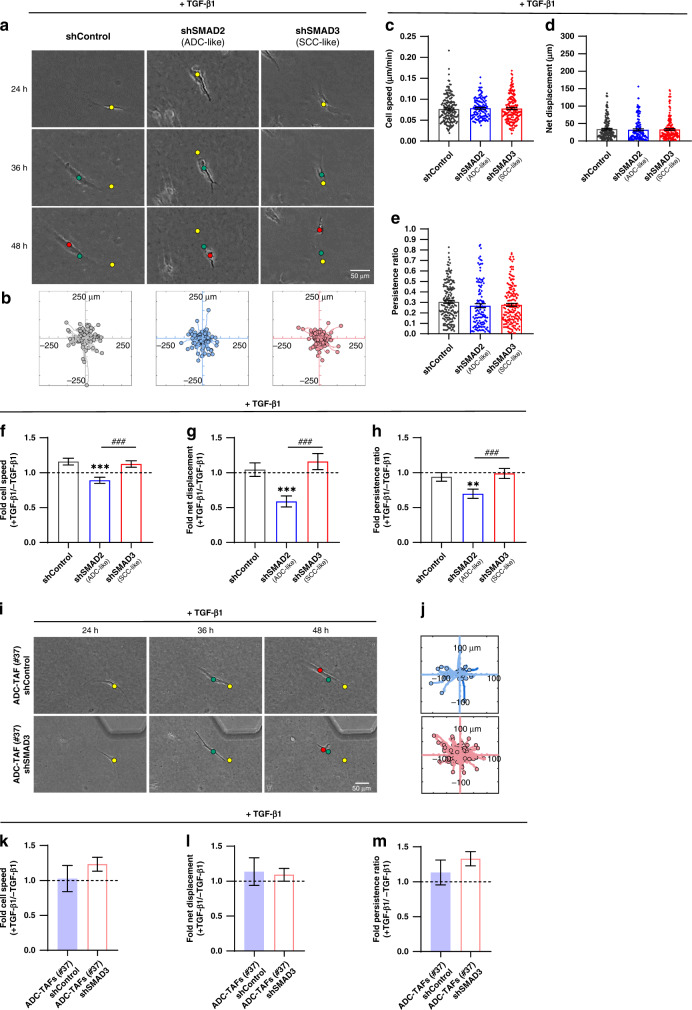
Impact of altered SMAD2/3 in 3D collagen migration in lung fibroblasts and ADC-TAFs stimulated with TGF-β1. **a** Representative phase contrast images of single control fibroblasts (#5) for each group (shControl, shSMAD2, shSMAD3) cultured in 3D collagen gels within the microdevice with TGF-β1 during the experimental time-window (24–48 h). Yellow, green and red dots indicate the position of the fibroblast centre at 24 h, 36 h and 48 h, respectively. Scale bar, 50 μm. **b** Trajectory maps corresponding to the tracking of the centre of fibroblasts from each group in basal conditions throughout the experimental time-window. **c**–**e** Average cell speed (**c**), net displacement (**d**) and persistence ratio (**e**) with TGF-β1 of single control fibroblasts (#5) for each group (shControl, shSMAD2, shSMAD3) gathered from three independent microdevices per condition (shown as bars). Each dot indicates the average of a single fibroblast. **f**–**h** Fold average cell speed (**f**), net displacement (**g**) and persistence ratio (**h**) assessed in the presence and absence of TGF-β1. Error bars were computed using error propagation [67]. **i** Representative phase contrast images of single shControl or shSMAD3 ADC-TAFs (#37) cultured as in **a**. Yellow, green and red dots indicate the position of the fibroblast centre at 24, 36 and 48 h, respectively. Scale bar, 50 μm. **j** Trajectory maps corresponding to the tracking of the centre of ADC-TAFs from each group with TGF-β1 throughout the experimental time-window as in **b**. **k**–**m** Fold average cell speed (**k**), net displacement (**l**) and persistence ratio (**m**) of ADC-TAFs (#37) for each group assessed in the presence and absence of TGF-β1. ^###^*p* < 0.005 comparing shSMAD2 and shSMAD3. **p < 0.01; ***p < 0.005 with respect to shControl or comparing shControl and shSMAD3 ADC-TAFs. Statistical comparisons were done using Student’s *t* test or Mann–Whitney (**c**–**e**). Mean values correspond to three independent microdevices.

The basal migratory advantage of shSMAD2 fibroblasts was abrogated upon TGF-β1 stimulation, and fibroblasts in all conditions exhibited similar trajectories (Fig. 4a, b), speed (Fig. 4c), net displacement (Fig. 4d) and persistence (Fig. 4e). Moreover, even though TGF-β1 barely reduced the average cell speed in shSMAD2 with respect to basal conditions (Fig. 4f), it elicited a marked reduction in the average persistence and associated net displacement (Fig. 4g, h), revealing that TGF-β1 promotes the spatial confinement of ADC-like fibroblasts. In contrast, shSMAD3 fibroblasts were poorly responsive to TGF-β1, exhibiting a small increase in speed and displacement compared to basal settings (Fig. 4f, g) in further agreement with protrusion analysis. A similar trend was observed in shControl compared to shSMAD3 ADC-TAFs (#37) in terms of speed and persistence (Fig. 4k–m); however, these differences did not attain statistical significance due to the large variability associated with their globally low speed and directionality. These results suggest that the spatial confinement of ADC-TAFs is evident even in the absence of exogenous TGF-β1.

The strong qualitative agreement between early protrusions (0–4 h) and subsequent migration data (24–48 h) encouraged us to conduct a correlation analysis between descriptors of both processes. Both migration persistence and net displacement were strongly and positively correlated (*R*^2^ > 0.7) with all protrusion descriptors but protrusion number (Supplementary Fig. 4E–L), with the highest correlations consistently observed with aspect ratio (Supplementary Fig. 4K, L). In contrast, we observed a poor correlation between migration speed and all protrusions descriptors (Supplementary Fig. 4A, D, G, J). These results further support the notion that those conditions that elicit fewer and longer protrusions may help polarise the cell along a major protrusion and facilitate directed movements, whereas increased number of protrusions is associated with reduced migration and subsequent spatial confinement [31, 36].

### The enhanced migration of high SMAD3 fibroblasts in basal conditions is independent of collagen degradation

**Fig. 5:**
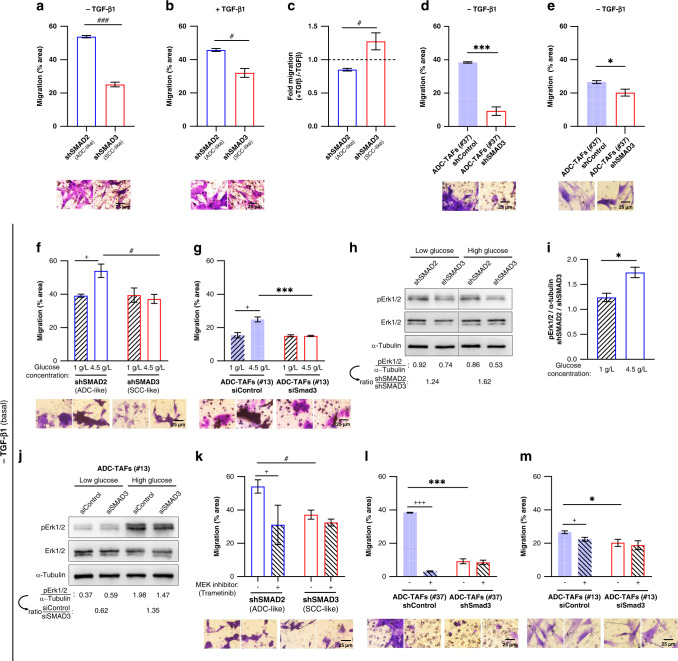
Mechanistic insights on the intrinsic basal migration priming of shSMAD3 fibroblasts and ADC-TAFs: role of high glucose, insulin–transferrin–selenium and Erk1/2. **a**–**c** Average migration assessed using the Boyden Transwell assay of shSMAD2 and shSMAD3 control fibroblasts (#5) in the absence (**a**) or presence of TGF-β1 (**b**), and corresponding fold values (**c**). The same plots including shControl fibroblasts are shown in Supplementary Fig. 5A, B. Bottom panels show representative images of the porous membrane of the Transwell insert containing the migratory fibroblasts stained with crystal violet at the end of the experimental time-window (16 h) henceforth. **d**, **e** Average Transwell migration of shControl versus shSMAD3 ADC-TAFs (#37) (**d**) and siControl versus siSMAD3 ADC-TAFs (#13) (**e**) in the absence of TGF-β1. *SMAD3* mRNA levels of siControl and siSMAD3 ADC-TAFs are shown in Supplementary Fig. 5C. **f**, **g** Average Transwell migration with low or standard high glucose concentration in either shSMAD2 and shSMAD3 control fibroblasts (#5) (**f**) or siControl and siSMAD3 ADC-TAFs (#13) (**g**) in the absence of TGF-β1 (i.e. basal conditions). Low glucose conditions started 3 days before seeding cells for the migration experiment. Basal average Transwell migration with low or high glucose in the presence or absence of insulin-transferrin-selenium (ITS) are shown in Supplementary Fig. 5F, G. **h**, **i** Representative Western blot analysis of phosphorylated Erk1/2 (pErk1/2), total Erk1/2 and loading (α-tubulin) of shSMAD2 and shSMAD3 fibroblasts cultured as in **f** and examined 30 min after seeding. Corresponding densitometric values of pErk1/2/α-tubulin are shown at the bottom (**h**) thereafter. Average densitometry ratio of pErk1/2 / α-tubulin in shSMAD2 with respect to shSMAD3 are shown in **i**. **j** Western blot of pErk1/2, total Erk1/2 and loading of siControl and siSMAD3 ADC-TAFs (#13) cultured as in **h**. Densitometry ratio of pErk1/2/α-tubulin in siControl with respect to siSMAD3 is shown at the bottom. **k**–**m** Average Transwell migration with or without the MEK1/2 inhibitor Trametinib (100 nM) in either shSMAD2 or shSMAD3 control fibroblasts (#5) (**k**), shControl and shSMAD3 ADC-TAFs (#37) (**l**) or siControl and siSMAD3 ADC-TAFs (#13) (**m**) in the absence of TGF-β1. Error bars represent mean ± s.e.m. ^#^*p* < 0.05; ^###^*p* < 0.005 comparing shSMAD2 and shSMAD3. *p < 0.05; ***p < 0.005 with respect to shControl. ^+^*p* < 0.05; ^+++^*p* < 0.005 comparing either low and high glucose or the presence and absence of Trametinib. Statistical comparisons were done using Student’s *t* test. Mean values correspond to *n* ≥ 2 experiments.

Because our migration analysis was conducted in fibroblasts embedded in a dense collagen matrix with a expected range of pore sizes comparable or even lower than the typical width of elongated fibroblasts [37], it is possible that the enhanced basal migration of high SMAD3 fibroblasts is driven by increased collagen degradation rather than intrinsic migratory priming. To address this question, we analysed migration in the complete absence of exogenous extracellular matrix (ECM) degradation using the Boyden Transwell assay without any ECM coating in the porous Transwell insert membrane. In agreement with our 3D migration data, the percentage of cells that migrated through the insert membrane was markedly higher in shSMAD2 fibroblasts compared to shSMAD3 in basal settings (Fig. 5a and Supplementary Fig. 5A). Likewise, the migration advantage of shSMAD2 fibroblasts was attenuated by TGF-β1, whereas shSMAD3 fibroblasts were poorly responsive (Fig. 5b, c and Supplementary Fig. 5B). Consistent differences were observed in ADC-TAFs from randomly selected patients (#13, #37) in which SMAD3 had been knocked down by shRNA (#37) (Fig. 3g) or siRNA (#13) (Supplementary Fig. 5C) compared to parental cells in Transwells in the absence of TGF-β1 (Fig. 5d, e). Likewise, migration of ADC-TAFs in Transwells was globally lower than control fibroblasts as in 3D. In contrast, we did not observe significant differences in the mRNA levels of the major collagenase MMP1 between either ADC-TAFs and SCC-TAFs from randomly selected patients (Supplementary Fig. 5D) or between shSMAD2 and shSMAD3 fibroblasts (Supplementary Fig. 5E). These findings reveal that the enhanced basal migration of high SMAD3 fibroblasts is largely independent of collagen degradation but rather due to an intrinsic priming of their migratory properties.

### The larger migration of high SMAD3 fibroblasts in basal conditions requires high glucose-dependent Erk1/2 hyperactivation

Prompted by the consistent migratory advantage observed in high SMAD3 conditions in both 3D collagen gels and Transwell assays selectively in the absence of TGF-β1, we began to explore the underlying mechanisms. A common exogenous factor in both assays was the presence of high glucose in the culture medium, which are standard conditions for the cell culture of fibroblasts and TAFs [38, 39]. Moreover, Erk1/2 can be activated by both high glucose and 3D collagen [40, 41], have been strongly implicated in the regulation of migration in numerous cell types including fibroblasts [42], and may interact with SMAD3 in the absence of TGF-β1 [43]. To examine the potential involvement of high glucose and/or Erk1/2 activation, we first analysed basal Transwell migration with either standard high glucose (4.5 g/l) or low glucose (1 g/l) medium and found a significant increased migration with high glucose in shSMAD2 but not in shSMAD3 conditions. In contrast, low glucose medium abrogated the migration differences between shSMAD2 and shSMAD3 fibroblasts (Fig. 5f). Likewise, Transwell migration was significantly increased in siControl ADC-TAFs (#13) in high versus low glucose conditions, whereas no differences were observed in siSMAD3 ADC-TAFs (Fig. 5g). Another supplemented soluble factor present in all assays was insulin-transferrin-selenium (ITS). Because a major function of insulin in cell culture is to stimulate glucose entry [44], we also analysed Transwell migration in the presence or absence of ITS and found a significant migration increase (~35%) with ITS in low glucose conditions selectively in shSMAD2 fibroblasts, whereas such migration increase was attenuated in high glucose conditions (~12%) (Supplementary Fig. 5F, G), further underscoring the requirement of high glucose in the basal migration priming of high SMAD3 fibroblasts. In contrast, shSMAD3 fibroblasts were consistently poorly responsive to both high glucose and ITS in terms of migration (Fig. 5f and Supplementary Fig. 5F, G).

Regarding Erk1/2 activation, we found that phosphorylated Erk1/2 (pErk1/2) levels normalised by α-tubulin were increased by ~70% in standard high glucose in shSMAD2 compared to shSMAD3 fibroblasts, whereas such increase was reduced to ~20% in low glucose conditions (Fig. 5h, i and Supplementary Fig. 5H, I). Likewise, normalised pErk1/2 levels were ~35% higher in siControl compared to siSMAD3 ADC-TAFs (#13) in our standard high glucose medium (Fig. 5j); however, this difference was smaller than that found between shSMAD2 and shSMAD3 fibroblasts, possibly due to the high endogenous expression of TGF-β1 in ADC-TAFs [9], which may alter pErk1/2 [45] and therefore bias the response to high glucose. In further qualitative agreement with migration data, normalised pErk1/2 levels were higher in shSMAD2 compared to shSMAD3 fibroblasts even in the absence of ITS (Supplementary Fig. 5J). On the other hand, total Erk1/2 levels remained fairly stable in all conditions (Fig. 5h, j and Supplementary Fig. 5I). These results unveil a SMAD3-dependent hyperactivation of Erk1/2 in the presence of high glucose.

To assess whether pErk1/2 hyperactivation is required for the basal migration priming of high SMAD3 fibroblasts, we analysed basal Transwell migration in the presence of 100 nM Trametinib, a clinically approved inhibitor of MEK1/2 MAP kinases in NSCLC and melanoma that acts right upstream of Erk1/2 [46]. Of note, Trametinib significantly downregulated basal migration in standard high glucose medium in shSMAD2 but not shSMAD3 fibroblasts (Fig. 5k). Consistently, Trametinib elicited a drop in basal Transwell migration in ADC-TAFs (#37, #13) in control conditions but not upon knocking down SMAD3 in the presence of standard high glucose (Fig. 5l, m). Yet, we noticed that the migration reduction elicited by Trametinib varied between ~20-80% depending on the cell model, even though Erk1/2 activation was strongly abrogated in all cases (Supplementary Fig. 5K, L), suggesting that additional molecular events other than pErk1/2 hyperactivation contribute to the migratory differences between high SMAD3 and high SMAD2 fibroblasts in high glucose conditions. Collectively, these results implicate high glucose-dependent Erk1/2 hyperactivation in the migratory advantage of high SMAD3 fibroblasts in the absence of TGF-β1, and reveal that high SMAD2 fibroblasts are poorly responsive to both high glucose/ITS and MEK1/2 inhibition.

### The enhanced basal migration of high SMAD3 fibroblasts is consistent with the larger accumulation of TAFs observed in ADC compared to SCC at early stages

**Fig. 6:**
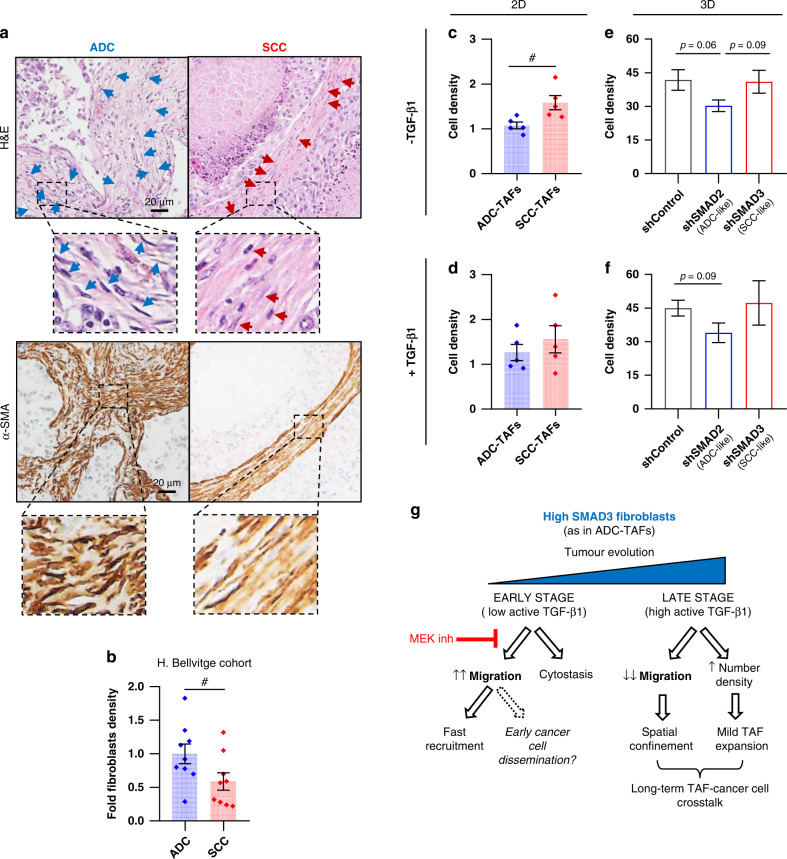
Differential TAF number density in ADC and SCC and potential contribution of SMAD2/3-regulated migration versus proliferation. **a** Illustrative haematoxylin–eosin (H&E) (top) and α-SMA (bottom) staining of ADC and SCC patients. Arrow heads point to scattered and spindle-shaped nuclei, which are histologic hallmarks of TAFs. **b** TAF number density assessed by morphometric analysis of haematoxylin images from the *Hospital de Bellvitge* patient cohort (10 ADC, 9 SCC). Independent validation with a smaller cohort is shown in Supplementary Fig. 6A. **c**, **d** Fibroblast number density of TAFs randomly selected from our cohort (5 ADC, 5 SCC) cultured in 2D in the absence (**c**) or presence (**d**) of TGF-β1. **e**, **f** Fibroblast number density of the control fibroblasts (#5) from all groups (shControl, shSMAD2, shSMAD3) cultured within the 3D collagen gels used to analyse migration in the absence (**e**) or presence (**f**) of TGF-β1. Corresponding values of fibroblasts cultured in 2D as in **c** are shown in Supplementary Fig. 6B, C. **g** Emerging model for the impact of the interplay between SMAD3, migration and proliferation in ADC-TAFs in early and late stages. Error bars represent mean ± s.e.m. Each dot corresponds to a different patient (**b**–**d**). ^#^*p* < 0.05; comparing either ADC-TAFs and SCC-TAFs or shSMAD2 and shSMAD3. Other p-values with respect to shControl. Statistical comparisons were done using Student’s *t* test. Mean values correspond to *n* ≥ 2 experiments.

Finally, we examined the potential contribution of our observed relationship between altered SMAD2/3 expression and migration to the excessive accumulation of TAFs in lung cancer, since we previously reported a larger TAF density in histologic samples in ADC compared to SCC patients [6] in a small patient cohort (5 ADC, 5 SCC) (re-analysed as number density/image field in Supplementary Fig. 6a). We confirmed this observation by assessing the number density of TAFs in an independent patient cohort (*Hospital de Bellvitge*; 10 ADC, 9 SCC), identified by their elongated nuclei (Fig. 6a top) and confirmed by their α-SMA expression (Fig. 6a bottom), and found consistent results (Fig. 6b).

TAF accumulation is thought to arise largely from the recruitment and/or proliferation of local resident fibroblasts [5, 47]. From the recruitment perspective, our observed migratory advantage without TGF-β1 in high SMAD3 fibroblasts is consistent with a larger recruitment of TAFs in ADC patients selectively at early stages, when active TGF-β1 is expected to be low [18]. To assess the potential contribution of differential proliferation, we examined cell number density—which is a common growth metric [5]—in different culture settings. Unlike histologic sections, ADC-TAFs exhibited a significantly lower number density in basal conditions compared to SCC-TAFs from randomly selected patients in 2D cultures (Fig. 6c). Likewise, basal number density was lower in shSMAD2 (ADC-like) fibroblasts compared to shSMAD3 both in 2D (Supplementary Fig. 6B) and in the same 3D cultures used in our migration studies (Fig. 6e). In contrast, all these differences were abrogated in the presence of TGF-β1 (Fig. 6d, f and Supplementary Fig. 6C). Moreover TGF-β1 consistently elicited a 10-20% increase in number density in ADC-TAFs (Fig. 6d) and shSMAD2 fibroblasts (Fig. 6f) compared to basal conditions, whereas such increase was not observed in SCC-TAFs, in agreement with our previously observed poor response of SCC-TAFs to soluble mitogenic cues [6]. Since we previously reported that differences in number density between ADC-TAFs and SCC-TAFs in 2D cultures were largely attributed to proliferation changes [6], our results support that altered SMAD2/3 expression elicits a proliferation advantage in terms of number density selectively in high SMAD2 fibroblasts as in SCC-TAFs in basal conditions that is not consistent with the larger TAF accumulation observed in ADC. Collectively these findings strongly support that the larger histologic TAF accumulation in ADC is driven, at least in part, by the enhanced migration of ADC-TAFs caused by their high SMAD3 expression at early stages, when active TGF-β1 is expected to be low [18], as summarised in Fig. 6g.

## Discussion

SMAD2/3 are important transcription factors of the canonical TGF-β1 pathway that are expressed in virtually all cell types [13, 45]. In fibroblasts, the TGF-β1/SMAD3 pathway has been extensively documented as a positive regulator of fibrosis in the lung and other organs [9, 14, 48]. In contrast, previous studies on the role of SMAD2/3 in fibroblast migration are scarce and limited to 2D cultures or transwells [15, 16], which do not capture the physiologic complexity of 3D microenvironments. To address these limitations, we used a microfluidic device to examine for the first time a panel of protrusions and migration descriptors of single fibroblasts in dense 3D collagen gels. Our gel density was >50% higher than that commonly used in other studies (1.5–2 mg/ml) [17, 29, 31, 49] to mimic the high collagen content reported in lung cancer patients [7]. In addition, we used a technical improvement in the protrusion analysis by checking different *Z* planes instead of a single *Z* plane as commonly reported [29, 36, 49].

We found that SMAD2/3 have a markedly distinct impact on migration depending on the TGF-β1 context. Specifically, our results revealed for the first time that high SMAD3 conditions (as in shSMAD2 fibroblasts and ADC-TAFs) provide a migratory advantage in terms of protrusions (fewer and longer) and subsequent migration (faster and more directional) compared to high SMAD2 conditions (as in shSMAD3 fibroblasts and SCC-TAFs) selectively in the absence of exogenous TGF-β1, whereas TGF-β1 markedly abrogated this migratory advantage, promoting the spatial confinement of fibroblasts. In contrast, high SMAD2 conditions were poorly responsive to TGF-β1 and consistently exhibited the largest number of protrusions concomitantly with the shortest protrusion length with and without TGF-β1, which were subsequently associated with impaired migration through less directional movement and a shorter net displacement. Consistently, downregulating SMAD3 in ADC-TAFs was sufficient to reduce basal migration in 3D and in Transwells. In agreement with our findings, TGF-β1 was associated with reduced migration in keratinocytes [50] and cardiac fibroblasts in Transwells [16]. Likewise, SMAD3 null cardiac fibroblasts exhibited a lower migration than wild-type cells upon 1% FBS stimulation in Transwells [16], in agreement with our high SMAD2 observations. In contrast, unlike our 3D findings, no migratory effects were observed in cardiac fibroblasts stimulated with 1% FBS upon knocking down either SMAD2 or SMAD3 with siRNA using Transwells [51], supporting that our multiparametric microdevice-based 3D migration analysis may be more sensitive in detecting migration changes in response to altered SMAD2/3 expression.

High SMAD3 fibroblasts were also the most sensitive to the presence of exogenous TGF-β1, exhibiting more and shorter protrusions that elicited a less directional movement and subsequently a shorter net displacement, thereby increasing their spatial confinement. Indeed, because TGF-β1 increased the number of fibroblast protrusions in all SMAD2/3 settings and it is known to increase traction forces in TAFs [52], it is likely that TGF-β1 elicits more simultaneous traction in different (random) directions, yielding an ineffective (non-persistent) movement despite holding or increasing cell speed. In line with this interpretation, ADC-TAFs exhibited lower basal migration than control fibroblasts, which is consistent with the higher basal secretion of TGF-β1 and expression of the contractility marker α-SMA [9, 53] in ADC-TAFs. In qualitative agreement with our observations, TGF-β1 did not promote migration in cardiac fibroblasts in Transwells [16], or even impaired it in keratinocytes [50]. In contrast, our results are not consistent with the TGF-β1 stimulation of in vitro wound healing reported in human vocal cord fibroblasts and keratinocytes or with its downregulation upon SMAD3 inhibition using a scratch assay [15, 54], since we observed either no migratory changes in 3D or even a moderate increase in Transwells in shSMAD3 fibroblasts in the presence of TGF-β1. However, the marked differences between the scratch assay and our 3D/Transwell assays may account for this discrepancy. Collectively, our results clarify the migration regulation of SMAD2/3 in fibroblasts in 3D and how it depends on the TGF-β1 context.

TGF-β1 inhibits growth through SMAD3 in numerous cell types including epithelial cells and keratinocytes [55–57]. In contrast, we observed cytostatic effects in high SMAD3 conditions in the absence of TGF-β1 only, whereas TGF-β1 elicited a moderate but consistent increase in number density particularly in high SMAD3 fibroblasts and ADC-TAFs. Likewise, a TGF-β1-dependent proliferation increase was reported in dermal fibroblasts [58], further supporting that TGF-β1 is not an effective cytostatic cytokine in fibroblasts. Although our mechanistic understanding of these TGF-β1-independent pro-migratory and cytostatic functions in high SMAD3 fibroblasts is limited, our results implicated high glucose-dependent Erk1/2 hyperactivation in the migratory advantage. Erk1/2 are important regulators of migration in numerous cell types [42], and we found that standard high glucose culture medium markedly increased both migration and pErk1/2 selectively in high SMAD3 fibroblasts compared to low glucose conditions. Conversely, the MEK inhibitor Trametinib consistently attenuated basal migration in high SMAD3 but not high SMAD2 fibroblasts. Our results are in agreement with previous work reporting high glucose-dependent Erk1/2 hyperactivation in kidney cells [41], and implicate for the first time SMAD3 in this hyperactivation and a subsequent migration enhancement in fibroblasts.

The molecular underpinnings of these new SMAD3-specific fibroblast functions remain to be determined. Yet, it is unlikely that they involve the C-terminal residues of SMAD3 that become phosphorylated in response to TGF-β1 as part of the canonical TGF-β1/SMAD3 pathway [59], since we previously showed that these residues remain unphosphorylated in the basal conditions (i.e. absence of TGF-β1) [9] in which the migration enhancement was observed. Alternatively, the linker region of SMAD3 could be implicated, since this region contains several phosphorylation sites that can be regulated by Erk1/2 and other kinases in the absence of TGF-β1 [43, 45], and there is growing evidence that the linker regions may modulate different cell functions in SMAD2 and SMAD3 independently of TGF-β1 [43, 60]. Moreover, it has been shown that high glucose enhanced the sensitivity to exogenous TGF-β1 in mouse embryonic fibroblasts and kidney epithelial cells [41, 61], and the SMAD3 linker was involved in this enhancement [41], in agreement with our observed strongest response to TGF-β1 selectively in high SMAD3 fibroblasts. On the other hand, it is worth noting that a larger glucose uptake was recently reported in ADC-TAFs compared to SCC-TAFs [62], which could also contribute to our observed high glucose-dependent Erk1/2 activation in high SMAD3 conditions as in ADC-TAFs. However, our current knowledge of the Erk1/2-SMAD2/3 crosstalk in the absence of TGF-β1 is still very scarce and warrants further investigations. Likewise, it would be interesting to assess how our observed high SMAD2-driven poor response to MEK inhibition in fibroblasts may contribute to the resistance to MEK inhibitors in cancer [63].

Our findings provide new insights on how TAFs may contribute to tumour progression in lung cancer depending on the TGF-β1 context and the histologic subtype. Under low active TGF-β1 conditions as in early tumour stages [18], the enhanced migration of high SMAD3 fibroblasts may be a major contributor to the larger TAF recruitment observed in ADC compared to SCC. In addition, because TAFs can promote cancer cell dissemination by leading collective cancer cell migration [64], it is conceivable that the basal migration priming of high SMAD3 fibroblasts may contribute to the early dissemination of cancer cells in ADC, which is a common clinical observation whose underlying mechanisms remain unknown [65]. However, testing this hypothesis awaits future investigations. Moreover, our results support that the tumour-promoting effects associated with the enhanced migration of high SMAD3 fibroblasts could be prevented with Trametinib or other MEK inhibitors in ADC but not SCC, since high SMAD2 fibroblasts as in SCC-TAFs were largely non-responsive to MEK inhibition. In contrast, at later stages where active TGF-β1 is expected to be abundant at doses within the same range used in our experimental settings [18, 21], the impaired migration observed in all SMAD2/3 conditions concomitantly with the expected α-SMA-dependent increased contractility may collectively increase the spatial confinement of TAFs and ultimately facilitate the long-term interactions with adjacent cancer cells to promote their epigenetic reprogramming [52, 66] (Fig. 6g).

In summary, we unveil how altered SMAD2/3 expression provides migration and proliferation advantages only in the absence of TGF-β1, although in opposite directions, since enhanced migration (but not proliferation) was observed selectively in high SMAD3 conditions, strongly supporting that the larger TAF accumulation in ADC occurs at early stages (under low active TGF-β1). Moreover, our results provide a rationale for the implication of ADC-TAFs in the early ADC cancer cell dissemination observed in clinical settings and for the therapeutic use of MEK inhibitors against the enhanced migratory phenotype of ADC-TAFs (Fig. 6g).

## Supplementary information

Supplementary Materials

Supplementary Figures

Supplementary Table

Reproducibility checklist form from journal

## Data Availability

The data sets generated and analysed in the study are available from the corresponding author on reasonable request.
