# Supplementary material for: 3D collagen migration patterns reveal a SMAD3-dependent and TGF-β1-independent mechanism of recruitment for tumour-associated fibroblasts in lung adenocarcinoma
Source: Br J Cancer. 2022 Dec 26;128(6):967–81. doi: 10.1038/s41416-022-02093-x (PMC10006167; doi:10.1038/s41416-022-02093-x)
Supplement: Supplementary file 1 — Supplementary Materials [file 41416_2022_2093_MOESM1_ESM.pdf]

SUPPLEMENTARY MATERIAL AND METHODS

3D collagen migration patterns reveal a SMAD3-dependent and TGF-β1-independent mechanism of recruitment for tumor associated fibroblasts in lung adenocarcinoma

Yago Juste-Lanas et al.

Histologic analysis

Tumor samples were processed as described (*1*), counterstained with haematoxylin and stained for the autophagy marker cleaved microtubule-associated protein 1 light chain 3 (LC3A) (#Ap1805a, Abgent), which is largely negative in fibroblasts (*2*). Histological stainings were imaged with a bright-field microscope (BX43) coupled to a digital camera (DP72) using a ×10 objective (Olympus, Japan). Fibroblast nuclear density was assessed by image analysis of haematoxylin staining using QuPath software (*3*) under the guidance of our pathologists (JR). Images were color deconvoluted and segmented using default parameters. Over detected cells, object classifier was trained with Random forest (Rtrees) supervised classifier to identify and count fibroblast nuclei, which were normalized by area and averaged for each patient (n=3 images/patient).

SMAD2 and SMAD3 knock-down with shRNA and siRNA

SMAD2 and SMAD3 were stably knocked down in immmortalized primary control fibroblasts from patient #5 with lentiviral vectors derived from pLKO.1-puro from Sigma MISSION collection: shRNA for SMAD2 (TRCN 0000010477) and SMAD3 (TRCN 0000330128), using the CalPhos Mammalian Transfection Kit (Clontech) following manufacturer´s protocol as reported (*1*). A nonmammalian targeting shRNA vector was used as control (SHC002), and was referred to as shControl. Briefly, HEK293T cells (ATCC CRL-3216) were transfected with suitable plasmids, and their supernatant containing lentivirus was filtered and used to transduce hTERT-immortalized fibroblasts. Transduced cells were selected with puromycin (Sigma) as described (*1*). SMAD3 was stably knocked-down in immortalized ADC-TAFs from patient #37 using the same protocol. Alternatively, SMAD3 was transiently knocked down in ADC-TAFs by siRNA. For this purpose, fibroblasts were seeded in complete medium overnight to reach 30-50% confluence. The following day, fibroblasts were transfected using Lipofectamine RNAiMAX (Invitrogen) and Silencer Pre-Designed siRNA constructs at 30 nM final concentration (control siRNA #4390843, siRNA SMAD3 ID: 107877, Thermo Fisher), following manufacturer's instructions. Cells were kept in culture medium for 48 hours for SMAD3 knockdown.

qRT-PCR

RNA extraction and reverse transcription were conducted as reported (*4*). *SMAD2/3* mRNA levels were assessed using specific primers for *SMAD2* (forward 5' GTC GTC CAT CTT GCC ATT CAC 3', reverse 5' TTC CTG CCC ATT CTG CTC TC 3'), *SMAD3* (forward 5' ATC CCA CCA GGA TGC AAC 3', reverse 5' TCA ACT GGT AGA CAG CCT CA 3') and *ACTB* (forward 5' CAA GGC CAA CCG CGA GAA GAT 3', reverse 5' CCA GAG GCG TAC AGG GAT AGC AC 3', used as an endogenous control) and SYBR Green Master Mix (ThermoFisher). *MMP1* mRNA was assessed using TaqMan Gene Expression Master Mix and TaqMan gene-specific primer pairs and probes for *MMP1* (Hs00233958_m1) and *POLR2A*

(Hs00172187_m1) (used as a housekeeping gene). Relative expression with respect to an endogenous control was computed as $2^{-\Delta Ct}$ (*5*).

Western blot (WB) analysis

WB analysis of SMAD2/3 was conducted as described (*1*). In brief, protein extraction was performed with a lysis buffer containing Tris 50 mM pH 7.4, NaCl 150 mM, SDS 0.1%, Triton X-100 1% (Sigma), Nonidet P-40 1% (Igepal), proteinase (Cocktail Set I, Merck; Pefabloc, Roche) and phosphatase (Phostop, Roche) inhibitors. Equal protein amounts were separated with precast gels, transferred to a PVDF membrane as described (*4*), blocked and incubated overnight with primary antibodies against total SMAD2/3 (#3102, Cell Signaling), pSMAD2 (#3104, Cell Signaling), pSMAD3 (#07-1389, Merck Millipore), Erk1/2, pErk1/2 (#9102 and #9101; Cell Signaling Technology), β-actin (#A1978, Sigma-Aldrich) and α-tubulin (#2144; Cell Signaling Technology). The latter two were used as loading controls. Protein bands were labelled, visualised by chemiluminescence (Imagequant LAS4000, GE Healthcare), and band intensities were analyzed with ImageJ (*6*) and normalized to the corresponding loading control.

Fabrication of the microfluidic device

Microfluidic devices were fabricated as described (*7*), using photomasks as previously reported (*8, 9*). In brief, masks (Micro Lithography Services) were used to fabricate positive 300 µm high SU8 masters (Stanford University), which were treated with $CF_3(CF_2)_5(CH_2)_2SiCl_3$ (Alfa Aesar) to facilitate subsequent detachment. Polydimethylsiloxane (PDMS) (Sylargd 184, Dow Corning) was mixed at a 10:1 weight ratio of base to curing agent and poured on the SU8 master until the desired thickness (4 mm) was obtained. The PDMS solution was degasified to eliminate air bubbles, cured in an oven, cutted out and removed from the wafer, perforated and autocleaved. PDMS microdevices were plasma-bonded to 35 mm glass-bottom petri dishes (Ibidi) and coated with 1 mg/ml poly-D-lysine (PDL) (Sigma-Aldrich) to enhance surface-collagen gel attachment. The geometry of the microdevice was based on (*10*) and included a 300 µm high central chamber to allocate the 3D collagen culture and two parallel liquid channels located on each side of the central chamber that were in direct contact with the gel for hydration and transport of nutrients and other factors (*9*).

Analysis of the migration of single fibroblasts in 3D collagen cultures

The relative displacement between images acquired at different time-points was corrected by image correlation using the device micropillars as reference points (*11*). Migration was analyzed blinded with respect to each group.

Fibroblast number density

Cell number density of TAFs in 2D cultures was assessed as reported (*12*). In brief, TAFs were cultured in serum-free medium with or without 2.5 ng/ml TGF-β1 for 5 days and their nuclei were stained with Hoechst 33342 (Molecular Probes) and imaged with an Eclipse TE2000 microscope (Nikon) at 9 randomized locations with an EM-CCD C9100a camera (Hamamatsu) using Metamorph software (Molecular Devices) and a 10× objective. Number density in the same 3D cultures used for 3D migration analysis were assessed by manually counting cells imaged at the end of the experiment in the best Z plane. For each experiment, number density was determined as the average cell density/image.

Statistical analysis

All experimental data are available from the corresponding author on reasonable request

SUPPLEMENTARY REFERENCES

1. R. Ikemori *et al.*, Epigenetic SMAD3 Repression in Tumor-Associated Fibroblasts Impairs Fibrosis and Response to the Antifibrotic Drug Nintedanib in Lung Squamous Cell Carcinoma. *Cancer Res* **80**, 276-290 (2020).
2. A. Giatromanolaki *et al.*, Prognostic relevance of light chain 3 (LC3A) autophagy patterns in colorectal adenocarcinomas. *J Clin Pathol* **63**, 867-872 (2010).
3. P. Bankhead *et al.*, QuPath: Open source software for digital pathology image analysis. *Sci Rep* **7**, 16878 (2017).
4. M. Gabasa, R. Ikemori, F. Hilberg, N. Reguart, J. Alcaraz, Nintedanib selectively inhibits the activation and tumor-promoting effects of fibroblasts from lung adenocarcinoma patients. *Br J Cancer* **117**, 1128-1138 (2017).
5. K. J. Livak, T. D. Schmittgen, Analysis of relative gene expression data using real-time quantitative PCR and the 2(-Delta Delta C(T)) Method. *Methods* **25**, 402-408 (2001).
6. M. D. Abramoff, P. J. Magelhaes, S. J. Ram, Image processing with ImageJ. *Biophotonics Int* **11**, 36:42 (2004).
7. Y. Shin *et al.*, Microfluidic assay for simultaneous culture of multiple cell types on surfaces or within hydrogels. *Nat Protoc* **7**, 1247-1259 (2012).
8. J. Plou *et al.*, From individual to collective 3D cancer dissemination: roles of collagen concentration and TGF-β. *Sci Rep* **8**, 12723 (2018).
9. N. Movilla, C. Borau, C. Valero, J. M. García-Aznar, Degradation of extracellular matrix regulates osteoblast migration: A microfluidic-based study. *Bone* **107**, 10-17 (2018).
10. W. A. Farahat *et al.*, Ensemble analysis of angiogenic growth in three-dimensional microfluidic cell cultures. *PLoS One* **7**, e37333 (2012).
11. R. M. Haralick, L. G. Shapiro, *Computer and Robot Vision*. (Addison-Wesley, 1992), vol. Vol II.
12. M. Puig *et al.*, Matrix Stiffening and beta(1) Integrin Drive Subtype-Specific Fibroblast Accumulation in Lung Cancer. *Molecular Cancer Research* **13**, 161-173 (2015).
