# Supplementary material for: 3D collagen migration patterns reveal a SMAD3-dependent and TGF-β1-independent mechanism of recruitment for tumour-associated fibroblasts in lung adenocarcinoma
Source: Br J Cancer. 2022 Dec 26;128(6):967–81. doi: 10.1038/s41416-022-02093-x (PMC10006167; doi:10.1038/s41416-022-02093-x)
Supplement: Supplementary file 2 — Supplementary Figures [file 41416_2022_2093_MOESM2_ESM.pdf]

SUPPLEMENTARY FIGURE 1.

A

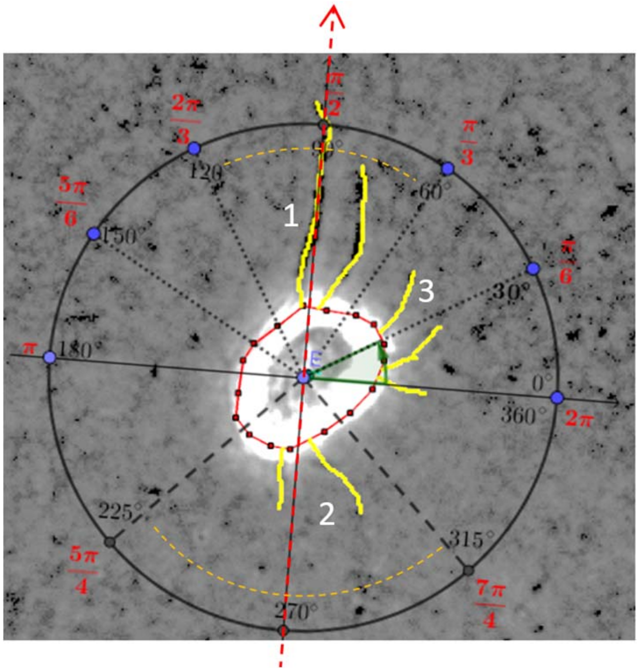

Supplementary Figure 1. Aspect Ratio, defined as major axis / minor axis, is calculated through the division of the length of the main axis by the length of the opposite longest axis. The longest protrusion is selected (1) creating an axis (red dotted line) towards the other side of the cell, through the centre of the cell (central blue dot). The longest protrusion of the opposite side (2) (< 45º deviation from the axis), together with the longest/major protrusion (1), and the cell body form the "major axis". The "minor axis" is formed by the longest protrusions of both sides of the major axis (excluding the opposite side of the main protrusion, and < 30º from the main protrusion, orange dotted lines). In this image, the minor axis is formed by the longest protrusion on the right (3) and the cell body, since there is no protrusion in the left side of the major axis.

SUPPLEMENTARY FIGURE 2.

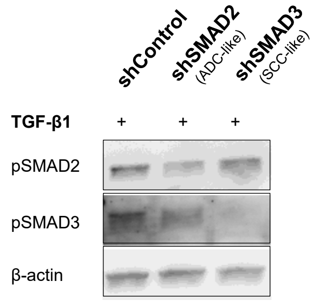

Supplementary Figure 2. Representative Western blot of phosphoSMAD2 and phosphoSMAD3 and β-actin of shControl, shSMAD2 and shSMAD3 control fibroblasts (#5) stimulated with 2.5 ng/ml TGF-β1 for 60 min.

SUPPLEMENTARY FIGURE 3.

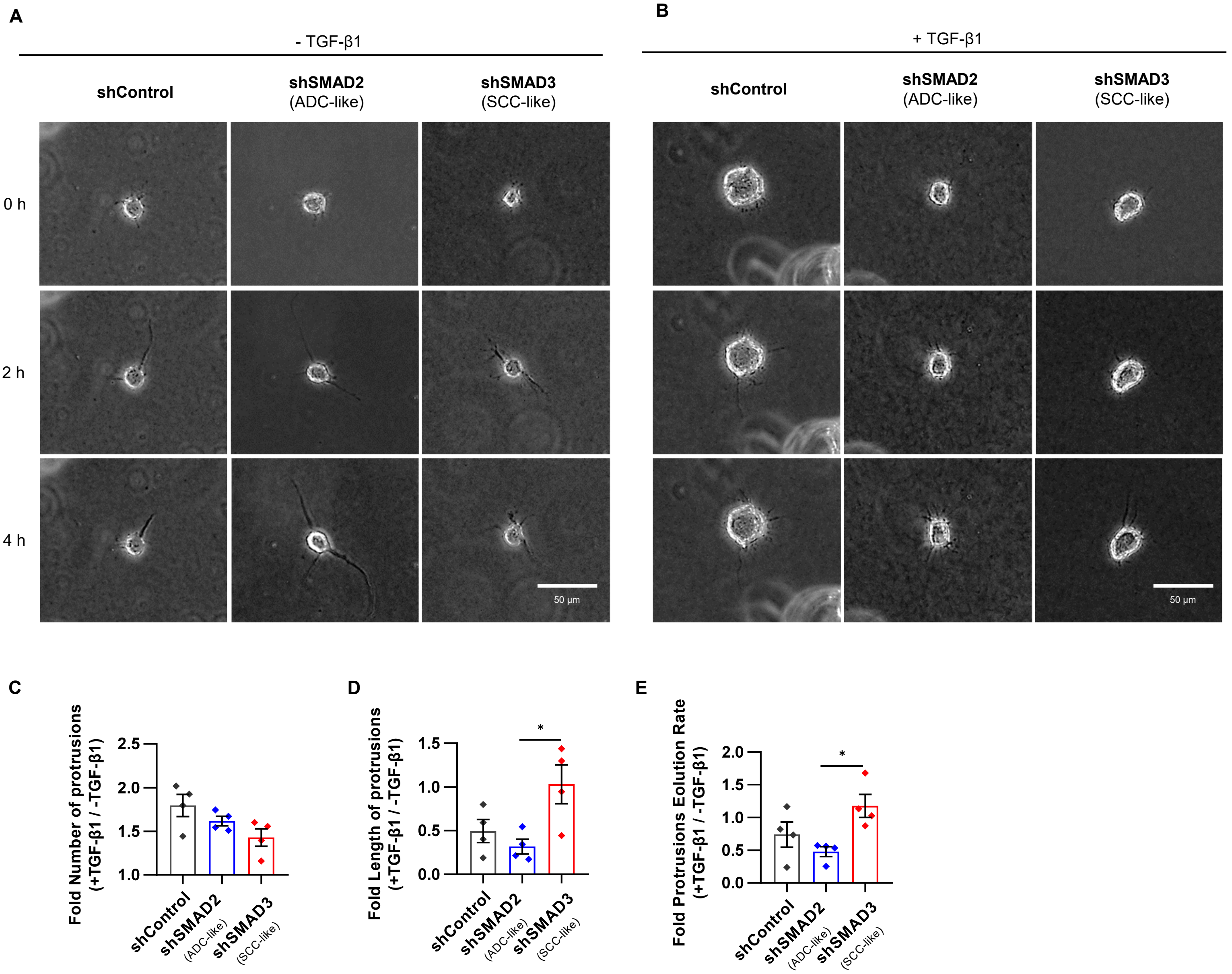

Supplementary Figure 3. Representative phase contrast images of single control fibroblasts (#5) for each group (shControl, shSMAD2, shSMAD3) cultured in 3D collagen gels within the microdevice for different times (0, 2 and 4h) in (**A**) basal conditions (-TGF-β1) or (**B**) treated with 2.5 ng/ml TGF-β1 (+TGF-β1). Scale bar, 50 µm. (**C-E**) Fold (+TGF-β1/ -TGF-β1) of the number of protrusions (**C**), protrusion length (**D**) and protrusions evolution rate (**E**) of 4 fibroblasts for each group cultured in 3D treated with 2.5 ng/ml TGF-β1 with respect to basal conditions. Error bars represent mean ± s.e.m. Each dot corresponds to a different fibroblast. *, $p < 0.05$. Statistical comparisons were done using Student's *t*-test.

SUPPLEMENTARY FIGURE 4.

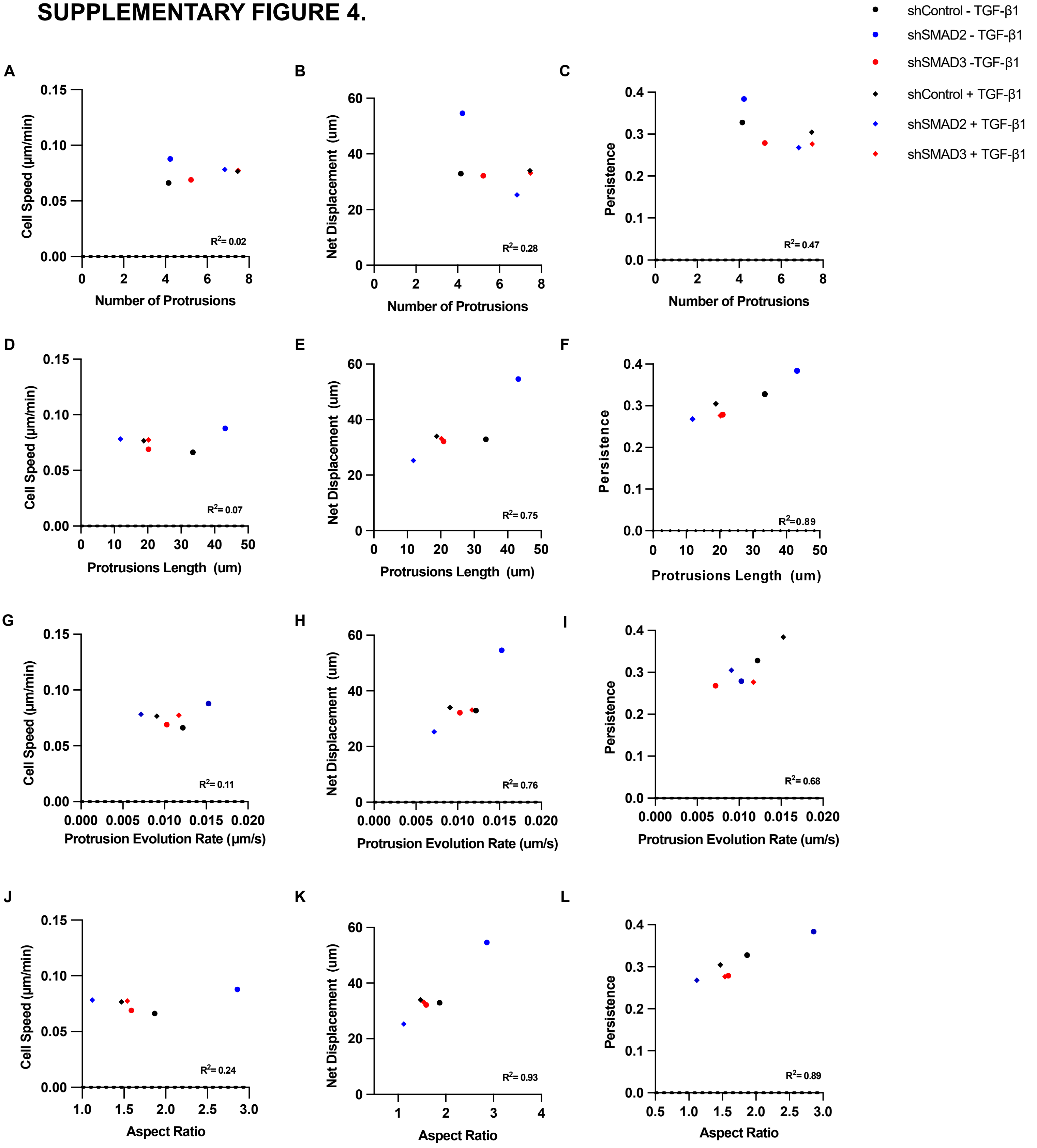

Supplementary Figure 4. Correlation between migration parameters (cell speed, net displacement and persistence) and protrusion parameters (number of protrusions (**A-C**), protrusion length (**D-F**) , protrusion evolution rate (**G-I**) and aspect ratio (**J-L**)) for each group (shControl, shSMAD2 and shSMAD3) in the absence or presence of TGF-β1. Pearson coefficient R^2 was calculated for each dataset.

SUPPLEMENTARY FIGURE 5.

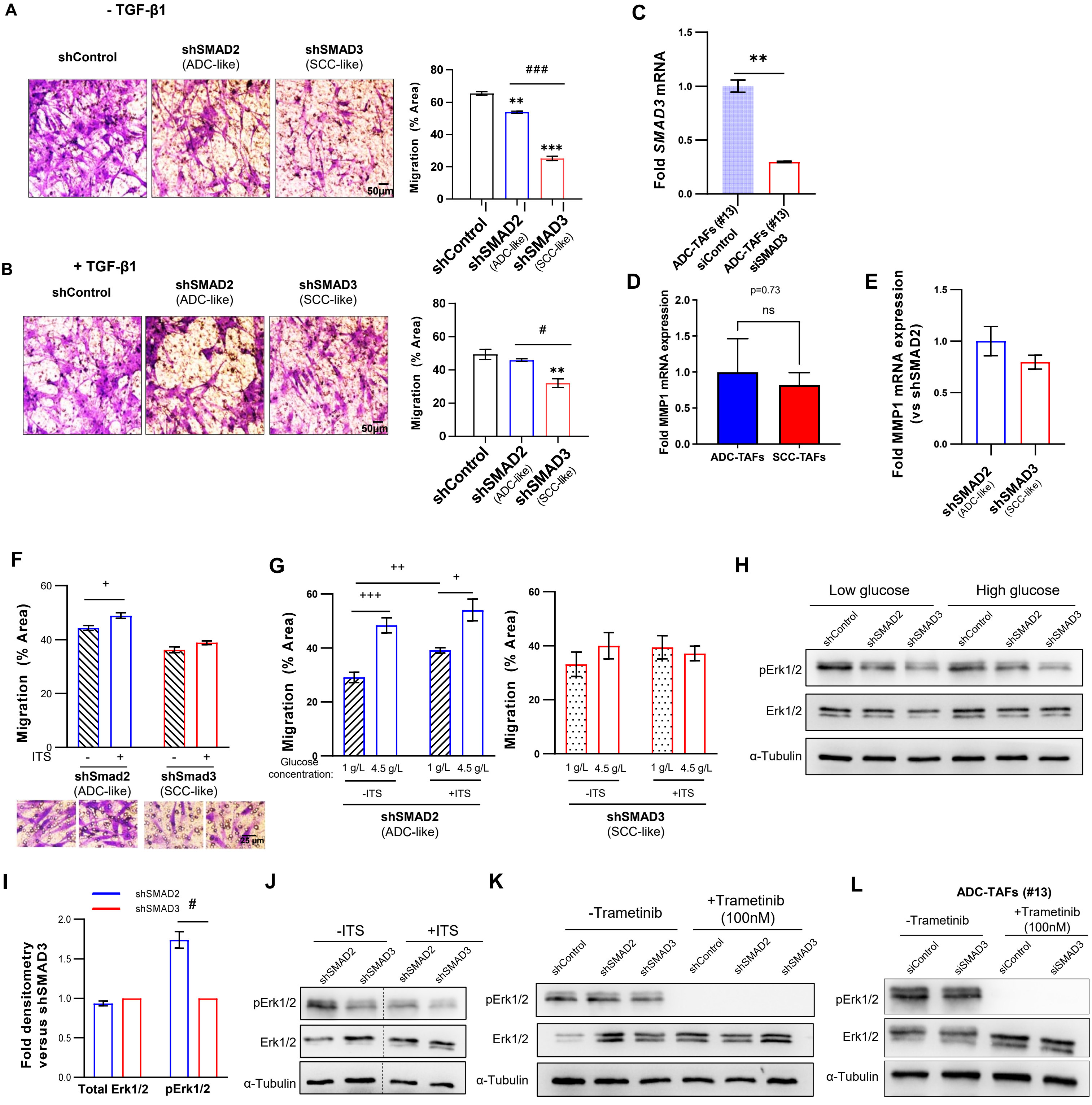

Supplementary Figure 5. Representative images and graphical representation of average migration assessed using the Boyden Transwell assay of shControl, shSMAD2 and shSMAD3 control fibroblasts CFs (#5) in (**A**) absence or (**B**) presence of TGF-β1. Fold SMAD3 mRNA of siControl and siSMAD3 ADC-TAFs (#13) (**C**). Basal *MMP1* mRNA levels of ADC-TAFs and SCC-TAFs (n=2) (**D**) and shSMAD2 and shSMAD3 CFs (**E**). Average Transwell migration with or without insulin-transferrin-selenium (ITS) in shSMAD2 and shSMAD3 control fibroblasts (#5) (**F**) and with high or low glucose conditions (**G**) in the absence of TGF-β1. Representative Western blot for total Erk1/2, pErk1/2 and α-tubulin of control fibroblasts under high or low glucose conditions (**H**) and corresponding densitometry analysis of shSMAD2/shSMAD3 in high glucose conditions (n≥2) (**I**). Representative Western blot for total Erk1/2, pErk1/2 and α-tubulin of shSMAD2 and shSMAD3 CFs (#5) with or without ITS (**J**). Representative Western blot of either shControl, shSMAD2 and shSMAD3 (#5) (**K**) or siControl and siSMAD3 ADC-TAFs (#13) (**L**) with or without trametinib. Error bars represent mean ± s.e.m. Statistical analysis as in main Fig5.

SUPPLEMENTARY FIGURE 6.

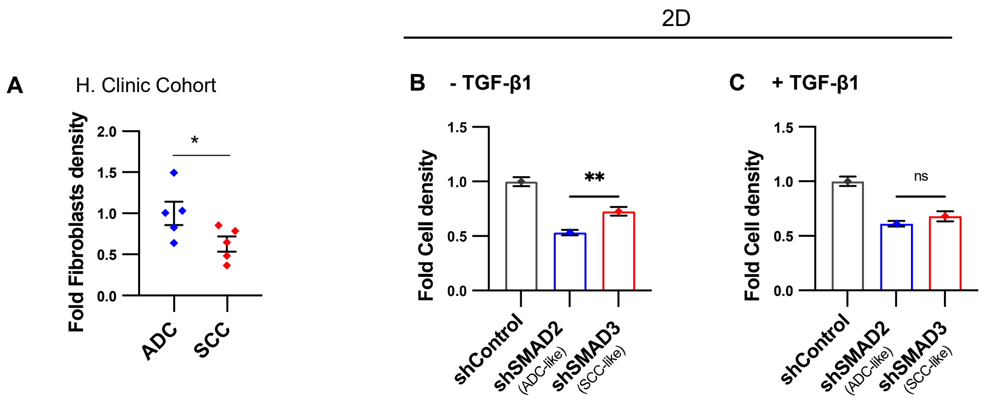

Supplementary Figure 6.(**A**) TAF number density assessed by morphometric analysis of haematoxilyn images from the *Hospital Clinic* patient cohort (5 ADC, 5 SCC). (**B-C**) Fibroblast number density of shControl, shSMAD2 and shSMAD3 cultured in 2D collagen-coated substrata in the absence (**B**) or presence (**C**) of TGF-β1. Error bars represent mean ± s.e.m. Each dot corresponds to a different patient. *, $p < 0.05$, **, $p < 0.01$. Statistical comparisons were done using Student's *t*-test.
