# Supplementary material for: 3D collagen migration patterns reveal a SMAD3-dependent and TGF-β1-independent mechanism of recruitment for tumour-associated fibroblasts in lung adenocarcinoma
Source: Br J Cancer. 2022 Dec 26;128(6):967–81. doi: 10.1038/s41416-022-02093-x (PMC10006167; doi:10.1038/s41416-022-02093-x)
Supplement: Supplementary file 3 — Supplementary Table [file 41416_2022_2093_MOESM3_ESM.pdf]

Supplementary Table 1: Summary of patient's clinical data

Juste-Lanas et al, *TGF-β1-independent priming of 3D collagen migration in high SMAD3 fibroblasts favours their early recruitment in lung adenocarcinoma*

***Hospital Clínic* cohort (used to obtain primary fibroblasts)**							
**Patient ref.**	**Age (y.o.)**	**Histologic subtype**	**Gender**	**Smoking history**	**pT**	**pN**	**Stage**
#2	82	SCC	male	n.a.	T3	N2	IIIA
#4	73	SCC	male	n.a.	T2b	N1*	IIB
#5	69	SCC	male	current	T2b	N2	IIIA
#6	65	SCC	male	former	T1a	N0	IA
#7	61	ADC	male	former	T1b	N0	IA
#10	76	ADC	male	former	T1a	N0	IA
#12	70	ADC	male	current	T3	N0	IIB
#13	59	ADC	male	former	T2b	N2	IIIA
#15	73	ADC	male	never	T1a	N0	IA
#16	64	SCC	male	former	T2a	N1*	-
#18	83	SCC	male	former	T2a	N0	IB
#20	78	SCC	male	current	T1b	N0	IA
#22	76	SCC	male	former	T2a	N1	IIA
#27	71	ADC	male	current	T1a	N0	IA
#28	80	ADC	male	former	T1a	N0	IA
#31	62	SCC	male	n.a.	T3	N0	IIB
#33	72	SCC	male	former	T2a	N0	IB
#34	76	SCC	male	n.a.	T2b	N0	IIA
#35	60	SCC	male	n.a.	T2a	N1	IIA
#37	58	ADC	male	current	T1b	N0	IA

N*: direct hilar ganglionar infiltration by the tumor

Hospital de Bellvitge cohort (used to assess TAF number density in histologic sections)							
**Patient ref.**	**Age (y.o.)**	**Histologic subtype**	**Gender**	**Smoking History**	**pT**	**pN**	**Stage**
#1B	61	ADC	male	former	T1B	N0	IA
#2B	59	ADC	male	current	T1A	N0	IA
#3B	69	ADC	male	never	T3	N0	IIB
#4B	80	ADC	male	former	T1B	Nx	IA
#5B	61	ADC	male	current	T3	N0	IIB
#6B	60	ADC	female	former	T2B	N0	IIA
#7B	51	ADC	female	current	T4	N2	IIIA
#8B	52	ADC	male	current	T1A	N0	IA
#9B	60	ADC	female	former	T2A	N0	IB
#10B	65	ADC	male	current	T4	N0	IIIA
#11B	58	SCC	male	former	T2A	N0	IB
#12B	76	SCC	male	former	T1B	N0	IA
#13B	72	SCC	male	current	T1A	N0	IA
#14B	60	SCC	male	current	T1A	N0	IA
#15B	69	SCC	female	never	T1A	Nx	IA
#16B	44	SCC	male	former	T3A	N1	IIIA
#17B	67	SCC	male	current	T1A	N0	IA
#18B	69	SCC	male	current	T1A	N0	IA
#19B	64	SCC	male	former	T3	N0	IIB
